# Transcriptome Changes during Major Developmental Transitions Accompanied with Little Alteration of DNA Methylome in Two *Pleurotus* Species

**DOI:** 10.3390/genes10060465

**Published:** 2019-06-17

**Authors:** Jiawei Wen, Zhibin Zhang, Lei Gong, Hongwei Xun, Juzuo Li, Bao Qi, Qi Wang, Xiaomeng Li, Yu Li, Bao Liu

**Affiliations:** 1 Key Laboratory of Molecular Epigenetics of the Ministry of Education (MOE), Northeast Normal University, Changchun 130024, China; wjw913@sina.com (J.W.); gongl100@nenu.edu.cn (L.G.); lijz346@nenu.edu.cn (J.L.); lixm441@nenu.edu.cn (X.L.); baoliu@nenu.edu.cn (B.L.); 2 Jilin Academy of Agricultural Sciences, Changchun 130033, China; xunhw334@nenu.edu.cn; 3 Engineering Research Center of the Ministry of Education (MOE) for Edible and Medicinal Fungi, Jilin Agricultural University, Changchun 130118, China; qibao3712@163.com (B.Q.); q_wang2006@126.com (Q.W.)

**Keywords:** *Pleurotus tuoliensis*, *P. eryngii*, DNA methylation, epigenetics, gene expression, development, interspecific divergence

## Abstract

*Pleurotus tuoliensis* (Pt) and *P. eryngii* var. *eryngii* (Pe) are important edible mushrooms. The epigenetic and gene expression signatures characterizing major developmental transitions in these two mushrooms remain largely unknown. Here, we report global analyses of DNA methylation and gene expression in both mushrooms across three major developmental transitions, from mycelium to primordium and to fruit body, by whole-genome bisulfite sequencing (WGBS) and RNA-seq-based transcriptome profiling. Our results revealed that in both Pt and Pe the landscapes of methylome are largely stable irrespective of genomic features, e.g., in both protein-coding genes and transposable elements (TEs), across the developmental transitions. The repressive impact of DNA methylation on expression of a small subset of genes is likely due to TE-associated effects rather than their own developmental dynamics. Global expression of gene orthologs was also broadly conserved between Pt and Pe, but discernible interspecific differences exist especially at the fruit body formation stage, and which are primarily due to differences in trans-acting factors. The methylome and transcriptome repertories we established for the two mushroom species may facilitate further studies of the epigenetic and transcriptional regulatory mechanisms underpinning gene expression during development in *Pleurotus* and related genera.

## 1. Introduction

*Pleurotus tuoliensis* (Pt) and *Pleurotus eryngii* (Pe) are two famous species of the *Pleurotus eryngii* complex that encompass the largest number of species in the oyster mushroom genus [1,2,3]. Both species are commercially important and widely cultivated especially in East Asia [4,5]. During sexual reproduction, basidiomycetes species undergo dramatic morphological changes driven by environmental factors such as temperature, photoperiod and culture substrates [6]. A previous study identified candidate genes related to mushroom formation in Pt, which was associated with reproductive growth, activation of specific transcription factors, upregulation of genes involved in the carbohydrate metabolism pathway and cold and light responses [7]. However, whole transcriptome analysis of Pe during developmental stages has not been reported. Moreover, with circa 18 million years divergence, significant morphological variations (especially in fruit body) have evolved between the two species [8]. Thus, an important question to ask is what are the genetic bases and molecular mechanisms underpinning the differences in growth habit and phenotypic transformation during important developmental transitions, i.e., from mycelium to primordium and to fruit body in the two mushrooms?

Compared with other forms of epigenetic modification (e.g., histone markers), DNA methylation is a relatively stable and heritable marker [9,10], DNA methylation exists in most animals, plants and fungi, and is involved in the regulation of diverse biological processes such as genomic imprinting, organ development, transposable elements (TEs) silencing and overall control of gene expression [11,12,13,14]. In mammals, DNA methylation is confined to the symmetric CG context, whereas in plants and fungi, it may occur at all cytosine bases classified into three sequence contexts, CG, CHG and CHH, where H = A, T or C [15,16,17]. In animals and plants, promoter and coding region of protein-coding genes often show moderate levels of DNA methylation (primarily in CG context), which associates with gene expression in either a negative or positive manner [11,15]. In contrast, it was found that the general landscape of CG methylation in representative fungi (belonging to ascomycete, basidiomycetes and zygomycetes) is largely depleted in genic regions and mainly enriched in TEs and other repetitive sequences [18]. This suggests that, in contrast to the situation in plants and especially animals, DNA methylation mainly retains the ancient function of TE silencing (the genome defense theory) in the fungal kingdom (*Eumycetes*). Recently, TE methylation has been further studied in Eumycota. For example, Montanini et al. found that incomplete TE methylation plays important roles in promoting genome plasticity [17]. Notably, TE-associated methylation contributing to silencing of adjacent genes was also demonstrated in several *Pleurotus* species [8,19,20].

Ample studies in human and animals have established that DNA methylation is dynamic across development. Genome-wide DNA methylation reprogramming occurred in mouse primordial germ cells and pre-implantation embryo [21,22]. In plants, methylome dynamics are also known to occur albeit to a lesser extent and/or confined to very specific cell types. For example, fruit ripening and endosperm development are accompanied with DNA hypomethyaltion [14,23,24,25]. Jeon et al. found that DNA methylation in both genic-regions and TEs also showed moderate dynamic changes during appressoria formation in the pathogenic fungus *Magnaporthe Oryza* [26]. Likewise, similar extent of methylation dynamics was observed during the sexual development of *Cordyceps militaris* [27]. In contrast, it was reported that gene expression change rather than methylation reprogramming triggers fruit body development in *Pleurotus ostreatus* [20].

Comparative transcriptome analysis is a powerful tool to unravel the genetic and molecular bases underpinning divergence of growth and development as well as differential environmental adaption between related organismal species. For example, it was found that enzymes involved in biomass degradation and related transcription factors were expressed divergently between two bagasse-degraded fungi, *Aspergillus niger* and *Trichoderma reesei* [28]. Orthologs involved in pathogenesis, including oxalate biosynthesis and endo-polygalacturonases, were differentially expressed between two plant pathogens, *Sclerotinia sclerotiorum* and *S. trifoliorum*, contributing to their different host ranges, although the two fungi are morphologically similar [29]. In basidiomycetes, comparisons among *Coprinopsis cinerea*, *Laccaria bicolor* and *Schizophyllum commune* indicated that most orthologs showed divergent expression at the fruit body stage [30]. 

In this study, we generated single-base resolution DNA methylomes by whole-genome bisulfite sequencing (WGBS) for the two commercially important *Pleurotus* species, *P. tuoliensis* and *P. eryngii*, at three major developmental stages, mycelium, primordium and fruit body, and we conducted comparative analyses. Our results suggest that major developmental transitions in the two mushroom species are associated with extensive changes of transcriptome but little alteration of DNA methylome.

## 2. Materials and Methods

### 2.1. Strains and Culture Conditions

All samples used for whole-genome bisulfite sequencing (WGBS) and RNA-seq were collected from identical batches of cultivated *Pleurotus eryngii* var. *eryngii* (Pe, strain ID: JKXB130DA) and *Pleurotus tuoliensis* (Pt, strain ID: JKBL130LB) at the three major developmental stages: mycelium, primordium and fruit body. Both monokaryotic mycelia were cultivated in PDA (Potato Dextrose Agar) liquid medium in dark at 23 °C for 7 days with shaking culture (120 rpm). Mycelium filtered by sterile gauze was ready for nucleic acid extraction. In order to acquire primordium, first, liquid cultures were transformed to cultivation bottles and grown in dark at 25 °C for 60 days with 70% humidity. For Pt, cold simulation was performed at 0 °C for 48 h. Then, cultivation bottles of Pt and Pe were under blue light condition (300lx–1000lx) at 14 °C for primordium initiation. After 10 days of cultivation, primordia with 2–3 cm were sampled from the bottles. Finally, the remaining cultures were transferred to a light (15 °C)/dark (7 °C) photoperiod of 12 h condition for about 15 days to induce fruit body formation. For RNA-seq, three biological replicates per condition were separately sampled. All samples were stored in liquid nitrogen until DNA and RNA extractions. Morphology of all sequencing samples were shown in Figure S1. 

### 2.2. Whole-Genome Bisulfite Sequencing and Data Processing

Genomic DNA (gDNA) from mycelium, primordium and fruit body of *P. tuoliensis* and *P. eryngii* was extracted by CTAB method and sheared by sonication to the size range of 200 to 300 bp. Library construction and Bisulfite treatment were processed as described in Hu et al. [31]. Sequencing was performed on the Illumina Hiseq 2500 platform (Illumina; San Diego, USA) with standard protocols. For data processing, first, Trimmomatic [32] was used to remove low-quality sequencing reads. Then, the clean data were aligned to the corresponding draft genomes of Pt and Pe with 1-bp mismatch by Bismark program [33]. Only uniquely mapped reads were used for further analysis.

### 2.3. Differential Methylation Analysis

Cytosine sites in CG-contexts with more than four uniquely mapped reads were used to further analysis. To decide methylated site, bisulfite non-conversion rate of 0.3% evaluated from unmethylated λDNA was used as background methylation level. Then, binomial test was performed for each cytosine site to decide if the observed methylation level significantly higher than such background methylation level [34,35]. P-values of the above tests were then adjusted by Benjamini-Hochberg method to q-values. Cytosine sites with q-value lower than 0.01 and corresponding methylation level larger than 5% were defined as methylated CG context (mCG). Differentially methylated regions (DMRs) between adjacent developmental stages were identified as described in [14] with minor modification. A sliding-window approach with 1kb size with step size of 200-bp was used to identify DMRs. Only windows including at least 10 mCG in at least one tissue were for further DMRs identification. For each window, Fisher’s exact test was performed to measure the difference of methylation level (reads count as agent) between adjacent developmental stages. P-values of above test were adjusted by Benjamini-Hochberg method to q-values. Windows with a q-value < 0.01 and the changes of methylation level ≥ 0.15 were defined as DMRs. Overlapping window with identical DMRs characteristics (hyper- or hypo-methylation) were merged into a large region.

Similarly, promoters (upstream 1kb of genes) and gene bodies including at least 10 mCG/kb in at least one tissue were defined as methylated promoter (MP) and methylated gene body (MGB), respectively, and were used for further identification of different methylation level described as above.

### 2.4. RNA-seq and Data Process

mRNA extracted from mycelium, primordium and fruit body of *P. tuoliensis* and *P. eryngii* were sequenced by the Illumina Hiseq 2500 platform. Raw data were filtered by removing low-quality reads. Filtered reads were aligned to each draft genome by Hisat2 [36] with default parameters. Read with uniquely mapped gene or transposable element (TE) (annotation as described in [8]) were kept for further analysis. The raw counts matrix was extracted by Stringtie with the provided python script (http://ccb.jhu.edu/software/stringtie/dl/prepDE.py) and DESeq2 [37] was used to identify differentially expressed genes (DEGs; q-value < 0.01 and log-transformed fold change >1). Gene and TE with TPM (Transcripts Per Million reads) large than 1 were taken as expressed. Gene expression information of both Pt and Pe were given in Supplementary dataset. for Single copy ortholog pairs between Pt and Pe were identified by OrthoMCL [38] after performing BLASTP with e-value < 1 × 10^−5^. After aligning pairwise by BLAT [39], orthologs with the proportion of consensus region larger than 70% and the length of consensus region > 300 bp were reserved for further analysis.

All the clean data including both WGBS and RNA-seq data have been deposited at the SRA data base http://www.ncbi.nlm.nih.gov/sra/ under accession number of PRJNA548464. 

### 2.5. Gene Ontology (GO) Enrichment Analysis

To trace the biological process of differentially regulated orthologs, GO enrichment analysis were performed by using Clusterprofiler [40]. Go terms with q-value < 0.05 and including at least five genes (orthologs) were deemed as significantly enriched.

### 2.6. Statistics

All Statistical tests in this paper were performed using basic packages in R language (Version 3.4.3, https://www.r-project.org/).

## 3. Results

### 3.1. DNA Methylation Landscapes in the Two Mushroom Species

**Figure 1:**
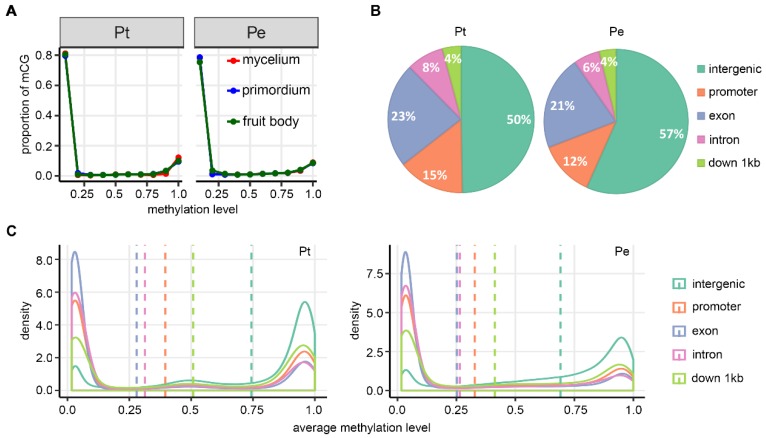
Landscape of DNA methylation in the two mushroom species, *Pleurotus tuoliensis* (designated Pt) and *P. eryngii* var. *eryngii* (designated Pe). (**A**) Distribution of DNA methylation levels of all covered CG sites (each having at least five reads) in all three tissues, mycelium, primordium and fruit body, in Pt and Pe. (**B**) Distribution of methylated CG sites (mCGs), in different categories of genomic features, intergenic, promoter, exon and down-1kb, in Pt and Pe. (**C**) Density of mCG levels among the three tissues belonging to different categories of genomic features in Pt and Pe. Dashed lines denote the average methylation levels of different genomic features in each species.

We generated single-base resolution DNA methylomes by whole-genome bisulfite sequencing (WGBS) in *P. tuoliensis* and *P. eryngii*, at three developmental stages, mycelium (MY), primordium (PR) and fruit body (FB). We focused on DNA methylation profiles of CG context only because of its predominance in the two *Pleurotus* species [8]. We found from 60% to 78% of CG contexts with sufficient sequencing depths (at least five reads at a given stage) were common between the two mushroom species across all stages. Methylation of these common CG contexts with high reliability were chosen to construct CG-context DNA methylation landscapes. We defined methylated CG contexts (mCGs) as those with q-values lower than 0.01 and methylation levels higher than 5% (Materials and methods). Based on this criterion, 22.3% (1.24 M) and 19.0% (0.99 M) CG contexts were mCGs in Pt and Pe, respectively, across the stages. We found that different developmental stages showed similar mCG levels in both species (16.0%, 15.8% and 15.6% in Pt; 16.5%, 16.2% and 16.9% in Pe). Similar to previous reports in fungi [17], mCG levels displayed a typical bimodal distributions in both mushrooms. Of all mCGs, 80.4% (Pt) and 77.4% (Pe) showed mCG levels lower than 10%, whereas the remaining showed levels > 70% (Figure 1A). More than 50% of mCGs were located in intergenic regions wherein TEs abound (Figure 1B). Typical bi-modal distribution of mCGs was observed in all types of genomic features (intergenic region, promoter, exon, intron and downstream 1kb of gene body) (Figure 1C). For intergenic regions enriched in TEs, the primary peak of mCGs was located in high methylation regions; whereas the primary peak of gene-related features was located to low methylation regions (Figure 1C). Pt and Pe showed the same trend in mCGs differences among the genomic features, i.e., both were intergenic region > gene downstream 1kb region > promoter region > exon = intron (Games-Howell post-hoc test).

We next investigated possible dynamics of mCG levels across the three developmental stages in both species. Differentially methylated regions (DMRs) were identified in both developmental transitions, transition 1 (from mycelium to primordium) and transition 2 (from primordium to fruit body). We found Pe possessed more DMRs than Pt in both transitions, although both mushrooms showed more DMRs in transition 1 than in transition 2 (Table S1; 180 versus 278 for Pt and 421 versus1428 for Pe; binomial test, *p*-values < 0.05). Intriguingly, the number of hyper-DMRs was more than that of hypo-DMRs in transition 2 in both mushrooms (binomial test, *p*-value < 0.05). For both hyper- and hypo-DMRs, intergenic regions possessed the highest proportions although they were also of variable ratios (54–70% for hyper-DMR and 77–83% for hypo-DMR, respectively; Figure S2). These results indicate biased distribution of mCGs in both Pt and Pe (enriched in TE regions and depleted in genic regions). Moreover, changes of mCG levels in the DMRs were apparent with developmental progression (phase 2 > phase 1), which again mainly occurred in TE-enriched intergenic regions.

### 3.2. Transcriptional Regulation of Gene Expression in the Two Mushroom Species

**Figure 2:**
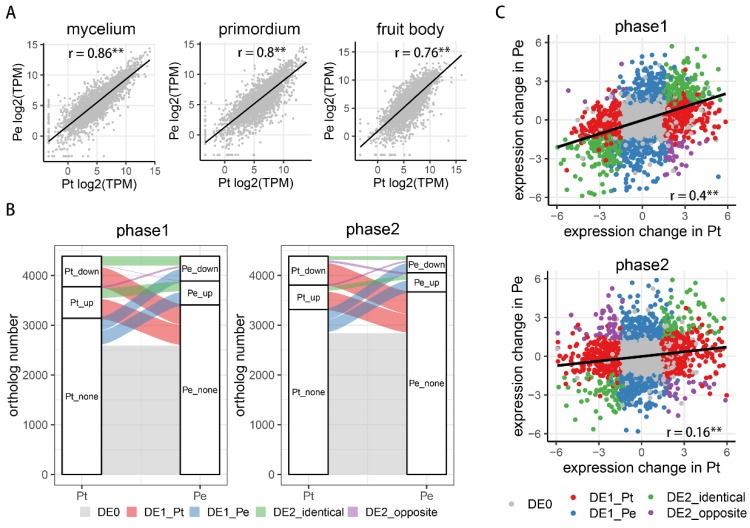
Expression changes between *Pleurotus tuoliensis* (Pt) and *P. eryngii* var. *eryngii* (Pe) in phase 1 and phase 2. (**A**) Correlation of absolute ortholog expression levels (TPM) between Pt and Pe at different developmental stages. (**B**) Alluvial diagram of orthologs expression change between Pt and Pe in each phase. Down, up and none indicating down-regulated expression, up-regulated expression and no change in expression, respectively. Alluvial with different colors represent different classes of genes. DE0: no expression changes in both orthologs; DE1-Pt: ortholog with expression change in Pt; DE1-Pe: ortholog with expression change in Pe; DE2-identical: both orthologs with expression change, identical trend; DE2-opposite: both orthologs with expression change, opposite trend. (**C**) Correlation of orthologs expression changes between Pt and Pe in each phase. The x-axis represents log2-transformed fold change between adjacent stages in Pt; the y-axis represents log2-transformed fold change between adjacent stages in Pe. The meaning of legends is same as (**B**). Asterisks in (**A**) and (**C**) indicate the *p*-value < 0.01 by Pearson’s product-moment correlation.

To investigate profiles of gene expression regulation in the two mushroom species (Pt and Pe), 4360 expressed single-copy orthologs (Materials and Methods) were identified and used to compare expression changes at the two developmental transitions or phases (phase 1, from mycelium to primordium; phase 2, from primordium to fruitbody) in both species. As shown in Figure 2A, orthologs between the two species exhibited well-correlated patterns of gene expression at the three stages (Pearson r = 0.75−0.84), which is similar with previous studies in plant species [41]. However, we note that the correlation of expression levels between the stages were significantly lower than those between biological replicates in each stages of the same species (Table S2), suggesting gene expression changes with development. Moreover, the correlations between gene expression decreased with progression of development.

**Figure 3:**
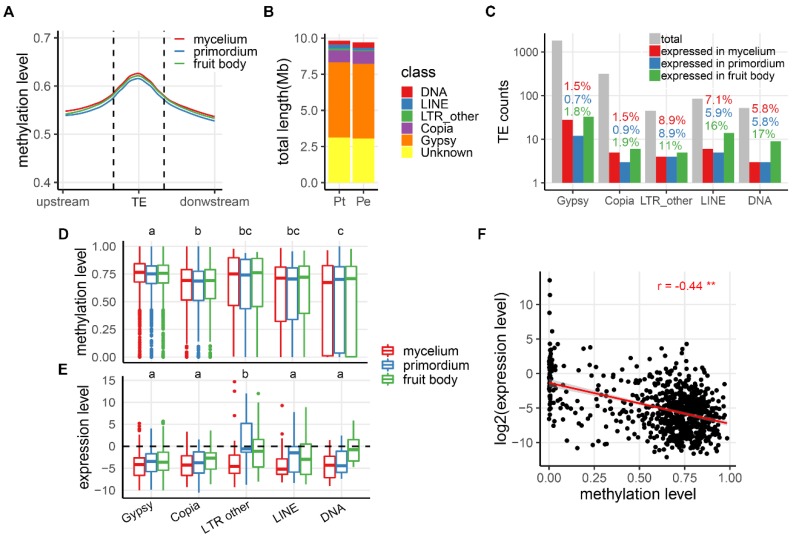
mCG and expression levels in transposable elements (TEs) of *Pleurotus tuoliensis* (Pt). (**A**) Meta-plots of mycelium (MY, red), primordium (PR, blue) and fruit body (FB, green) mCG levels in TEs. (**B**) Distribution and relative proportion of different classes of TEs. (**C**) Number of TEs in each class (grey bars) and number of expressed TEs (TPM > 1) in the MY, PR and FB stages. Percentages above the bars showing proportions of expressed TEs in each class. (**D**,**E**) mCG levels (**D**) and expression levels (**E**) for each class of TEs in the M, P and F stages. The dashed line in (**E**) indicates the static expression trend. The letters above the boxes denote results of Post Hoc Multiple Comparisons among the different developmental stages, wherein different letters denote statistical significance. Only TEs with a TPM value > 0 are shown in (**E**). (**F**) Negative correlation between mCGs and expression of TEs. Only TEs with a TPM value > 0 are tabulated. Very similar profiles were obtained for Pe, shown in Figure S4.

To compare the divergence of expression changes between species in the two phases, five classes of genes were defined: DE0 (non-differentially expressed between adjacent stages), DE1-Pt (differentially expressed in Pt), DE1-Pe (differentially expressed in Pe), DE2-identical (differentially expressed in both Pt and Pe, with identical trend) and DE2-opposite (differentially expressed in Pt and Pe, with opposite trend). We found that most ortholog pairs (~69%, 2955 out of 4360) showed identical pattern of expression changes in both phases (including DE0 and DE2-identical classes), suggesting that majority of orthologs showed constant expression during fruit-body formation in both mushrooms (Figure 2B). Around 18.7% (813 out of 4360 in phase 1 and 814 out of 4360 in phase 2) of orthologs belonged to DE1-Pt class in both phases, whereas 12.5% (545) and 10.8% (470) of these orthologs belonged to DE1-Pe in phase 1 and phase 2, respectively. Only 1.1% (47) DE2-oppsite orthologs occurred in phase 1, whereas the percentage was increased to 1.9% (82) in phase 2. GO analysis showed that orthologs with divergence of expression changes (including classes of DE1-Pt, DE1-Pe and DE2-opposite) were significantly enriched in the oxidation-reduction process in both phases (Table S3), indicating regulatory divergence of these genes between the two mushroom species may contribute to their morphological differentiation during development. Correlation of expression changes at each phase showed that regulatory difference of orthologs is more diverged in phase 2 than in phase 1 (correlation coefficient: 0.4 versus 0.16) (Figure 3C). Similar phenomenon also occurred when excepted orthologs belonged to DE0 class (correlation coefficient: 0.46 versus 0.19). Meanwhile, genes related to essential biological processes such as reproduction and oxidation reduction or are transcription factor and CAZYs were also chosen for correlation analysis. Except for genes related oxidation reduction, the remaining genes showed similar patterns to all orthologs, namely, expression change was more divergent in phase 2 than in phase 1 (Figure S3). Conversely, in both phases, genes related to oxidation reduction showed high divergence of expression changes, indicating these genes were diverged at earlier developmental stages between Pt and Pe. These results suggest the extent of expression divergence between the two mushroom species is magnified when formatting fruit body.

### 3.3. Correlation Between CG Methylation and Expression of TEs during Development in the Two Mushroom Species

TEs (including their derivatives forming other types of repeats) showed substantially higher mCG levels than genic regions in both species during in all three developmental stages (on average 0.55 and 0.52 in Pt and Pe, respectively) (Figure 3A; Figure S4A). We noted that the two mushrooms showed some differences in the changing trends of mCG levels in TEs across the developmental stages. In Pt, methylation profile in TEs was highest in mycelium, followed by fruit body, and lowest in primordium; whereas in Pe, it was highest in fruit body, followed by primordium and mycelium. In spite of these differences, mCG levels in TE regions differed by no more than 2% across the three developmental stages in both species.

To test for a possible relationship between expression of TEs and their mCG levels, well-annotated TEs were classified into five categories: *Gypsy*, *Copia*, other long-terminal retrotransposons (LTR-others), long interspersed nuclear elements (LINEs) and DNA transposons. As shown in Figure 3B, composition and content of TEs were very similar between the two mushroom species. In general, only small proportions of TEs were expressed during the three development stages (2.0%, 1.2% and 2.5% in Pt and 2.0%, 1.2% and 2.1% in Pe; Figure 3C and Figure S4B). The *Gypsy* and *Copia* retrotransposons, which represented ~62% of total repeat sequences in both species, showed the least proportions of expressed TEs among the different stages (on average, 1.3% of *Gypsy* and 1.1% of *Copia* in Pt; 1.3% of *Gypsy* and 2.2% of *Copia* in Pe), while relatively higher proportions of expressed TEs were found for the other three categories of TEs (9.6% of LTR-others, 9.8% of LINEs and 9.6% DNA transposons in Pt; 7.8% of LTR-others, 10.1% of LINEs and 4.3% DNA transposons in Pe). Notably, the fruit body stage showed the largest proportions of expressed TEs of all categories in Pt, whereas this phenomenon was seen only for *Gypsy*, LTR-others and DNA transposons in Pe. As shown in Figure 3D, the different categories of TEs showed minor difference of mCG levels in Pt: *Gypsy* was the highest methylated, whereas the other types of TEs showed similar mCG levels except for the comparison between *Copia* and DNA transposons (Games-Howell post-hoc test, *p*-value < 0.05). For each class of TEs, mCG levels were highly conserved across the three developmental stages. For Pe, all TE classes showed virtually identical methylation levels in all three developmental stages (Figure S4C). Similarly, generally low expression levels occurred in different classes of TEs in all three developmental stages (Figure 3E and Figure S4D). Nevertheless, when focusing on TEs with TPM > 0 (average levels among stages), a significant negative correlation between expression levels and mCGs was detected (Figure 3F; Figure S4E).

Although generally conserved mCGs across the different stages, small numbers of differential-methylated TEs (dmTEs) between adjacent stages did exist in both mushrooms. Relative to Pt, Pe possessed higher numbers of dmTEs (Figure S5A,C; Table S4). However, differential mCG levels in these TEs contributed little to their expression, as they remained largely repressed during development (Figure S5B,D; Mann-Whitney-Wilcoxon test, *p*-value > 0.05). This result suggests that, at least between adjacent developmental stages, the occurrence of minor changes of mCGs in TEs did not affect their generally low expression levels.

### 3.4. Correlation of CG Methylation and Expression in Protein-Coding Genes

**Figure 4:**
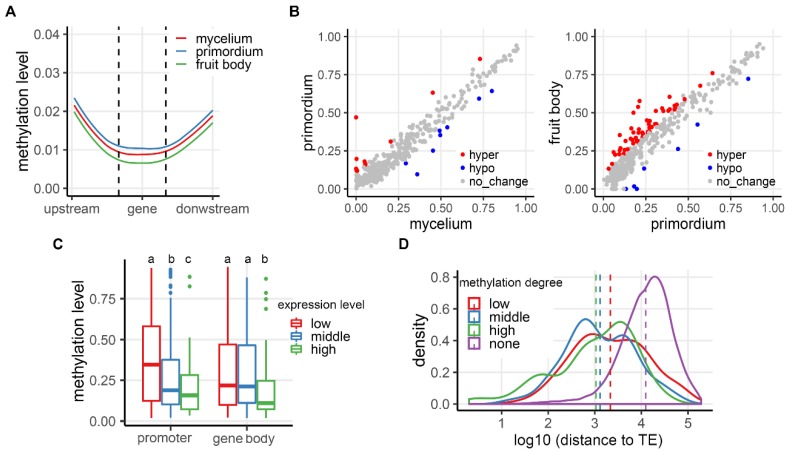
mCG and expression levels in protein-coding genes of *Pleurotus tuoliensis* (Pt). (**A**) Meta-plots of mycelium (M, red), primordium (P, blue) and fruit body (F, green) methylation levels in genic regions. (**B**) Correlation of mCG levels in promoter regions during transition 1 (from mycelium to primordium, left panel) and transition 2 (from primordium to fruit body, right panel). The red, blue and grey dots indicate hyper-methylated, hypo-methylated and unmethylated promoters, respectively. (**C**) Effects of mCG in promoters and gene bodies on gene expression (genes harboring methylated promoters or gene bodies were divided to low, medium and high expression classes). (**D**) Distribution of distances between different types of promoters (with respect to mCG) to their nearest TEs (none, low, medium, high mCG-containing promoters). Very similar profiles were obtained for Pe, shown in Figure S6.

We investigated potential changes in mCG levels across the developmental stages for the expressed protein-coding genes (expressed at least at one stage). In both mushrooms, the average mCG levels in the body region of protein-coding genes (gene body) and their flanking regions were generally very low across the three developmental stages (<3%). However, moderate differences were noted between the two mushroom species at one or more stages. Specifically, Pt showed similar methylation levels among the three developmental stages, whereas Pe showed relatively higher methylation levels in fruit body, although the between-stage differences remained less than 1% (Figure 4A and Figure S6A). To explore the potential correlation between mCGs and expression of protein-coding genes, we dissected genic regions into genes containing methylated promoters (MPs) and genes containing methylated gene bodies (MGBs) in each mushroom (Materials and Methods). More genes with MPs (722) and MGBs (365) were identified in Pe than in Pt (MPs and MGBs were 405 and 163, respectively). Strong correlations of mCG levels between adjacent developmental stages were detected for both groups of genes with MPs or MGBs in both mushrooms, suggesting mCGs in protein-coding genes were largely conserved during development (correlation coefficient > 0.9; Figure 4B and Figure S6B). However, clear differences of mCG levels were detected in each mushroom: (*i*) mCG level difference in MPs was elevated in transition 2 compared with transition 1 (14.8% versus 4.7% in Pt and 18.0% versus 6.8% in Pe, respectively); and (*ii*) the numbers of genes with hyper-MPs were significantly more than hypo-MPs in transition 2 (53 versus 7 in Pt and 125 versus 5 in Pe, respectively; Figure 4B and Figure S6B). MGBs showed a similar trend as MPs. However, similar to the situation of TEs, described above, for both MPs and MGB, small changes of mCG levels in protein-coding genes between the adjacent developmental stages contributed little to their expression changes (Figure S7; Mann-Whitney-Wilcoxon test, *p*-value > 0.05).

In light of the generally high methylation level of TEs, we suspected that a major role of DNA methylation in protein-coding genes was also to suppress rather than activate their expression during development. If this were the case, we would expect that the genomic environments (e.g., distance from TEs) wherein the genes residing should be a major deterministic factor. To test this, we first divided the promoter-methylated genes and gene body-methylated genes into three classes based on their averaged expression levels across the three developmental stages, and compared the DNA methylation levels among the gene classes. We found that in both Pt and Pe highly expressed genes tended to be less methylated in the corresponding promoters and gene bodies than lowly expressed genes (Games-Howell post-hoc test, *p*-values < 0.01; Figure 4C; Figure S6C). Consistent with this result, we found genes with unmethylated promoters were far away from their nearest TEs, followed by genes with promoters with medium and high methylation level, respectively (Figure 4D; Figure S6D). These results suggest that a major source of methylation in protein-coding genes in the two mushroom species likely stemmed from spreading from adjacent methylated TEs.

To investigate whether MPs and MGBs contributed to gene expression differences between the two mushrooms, differences in mCG levels and expression divergence between the 4452 single-copy ortholog pairs were quantified (Materials and Methods). We found that a total of 294 (6%) ortholog pairs containing MPs in either Pt or Pe and only 19 (~0.4%) ortholog pairs containing shared MPs between the two species (Figure S8A–C). Similarly, a total of 56 (1.2%) of ortholog pairs containing MGBs in either Pt or Pe and only one ortholog pair contained common MGBs (Figure S9A–C). Moreover, mCG divergence between promoters and gene bodies was not correlated with expression divergence of the corresponding orthologs (Figure S8D–J and Figure S9D–J; Mann-Whitney-Wilcoxon test, *p*-values > 0.05). This suggests that both MPs and MGBs have little contribution to the overall gene expression divergence between the two mushroom species at all three developmental stages, although we cannot rule out the possibility that the interspecific expression differences of a small subset of genes are regulated by differential MPs and/or MGBs.

## 4. Discussion

*Pleurotus toliensis* and *P. eryngii*, as two widely cultivated edible mushrooms, have been commercialized as nutritional, medicinal and animal feed products [42,43,44]. After ~18 MYA (Million Years Ago) divergence, the two species differ in aspects of habitat preference, growth condition, wood-decaying enzymes and fruit body phenotypes [3,8,45]. Major goals of this work were to establish the methylome landscapes and global gene expression profiles during major developmental transitions in the two mushroom species. We found that overall DNA methylation levels and patterns (CG methylome) are highly conserved between the two species and largely stable across the major developmental stages, while overall gene expression profiles (transcriptomes) manifest development-dependent interspecific divergence with fruit body showing the largest difference. Thus, our study provides important epigenome and transcriptome information in relation to development and divergence of the two mushroom species.

DNA methylation shows conserved properties, such as predominant confining to CG contexts, preferential localization in TEs, and largely depletion in genic regions, across the entire fungal phylum [18]. These properties of DNA methylation are fully recapitulated in our results. A remarkable observation is that both the content and composition of TEs are very similar between the two mushrooms, Pt and Pe, albeit their ~18 MYA divergence. In line with conservation of TEs, Pt and Pe showed similar methylation landscapes in both TEs and genic regions. This may suggest the high efficiency of DNA methylation as a genome defense system in *Pleurotus* [19]. Our results also accord with previous studies demonstrating the high conservation of global DNA methylation patterns in both ascomycetes and basidiomycete [17,18,26].

Our results show that mCG levels are static across major developmental transitions in both mushrooms, and which applies to both TEs and protein-coding genes. This lends further support to the previously proposed notion that, in contrast to animals and plants [14,21], DNA methylation appears to play limited developmental roles in the fungus kingdom [20].

We found that compared with TEs, expressed protein-coding genes contain very low levels of mCG in general. Nevertheless, still hundreds of genes were identified to contain methylated promoters in both mushrooms. For these genes, a significant negative correlation exists between mCGs and gene expression levels, suggesting that for a subset of genes, DNA methylation likely plays regulatory role in their expression in *Pleurotus*.

Previous studies indicate that genes adjacent to TEs tended to be transcriptionally silenced by DNA methylation [8,19,20]. In line with this observation, we also found that mCG levels of methylated promoters reduce gradually as distances to their nearest TE increase. We consider that it is possible that DNA methylation detected in genic regions of the two mushrooms species may have been the by-products derived from adjacent TEs rather than being modified via direct targeting. However, our current data cannot directly test this scenario, and therefore, we cannot rule out alternative possibilities such as some unique properties of the TE-adjacent genes (relative to TE-remote genes) for direct targeting by the methylation machinery. Irrespective of origin of the low level genic methylation in *Pleurotus*, our results do suggest that, when occurring, it plays some a role in regulating gene expression.

Fruit body formation involves complex developmental processes in basidiomycete fungi. Transition from vegetative mycelium to primordium requires the aggregation of cells into compact hyphal knots [46]. Then, fruit body is formed after a battery of cellular events including differentiation of primitive hymenium, karyogamy generation, stipe elongating and cap expanding [47]. Conceivably, these processes entail complex regulatory transcriptional rewiring to activate specific sets of genes, and the fact that the two mushroom species, Pt and Pe, are known to differ conspicuously in their fruit body morphology, suggesting major interspecific differences should also exist, and which entails further investigations.

Transcriptional rewiring can be due to changes in cis-elements (e.g., core regions of promoters or enhancers), trans-factors (e.g., diffusible transcription factors and their collaborating modifiers), and their interactions. As expected, we found that during developmental transitions in both mushrooms, trans-factors play a major role in regulating the differential gene expression profiles, while changes in both cis- and trans-factors appear to contribute to the interspecific differences in gene expression profiles between the two mushrooms.

A notable result from our study is that orthologs related to the oxidation-reduction process expressed most divergently between the two mushrooms especially at the fruit body formation stage. Previous studies also indicated that genes related to oxidation-reduction, such as those coding for carbohydrate-active enzymes (CAZy enzymes) and cytochrome P450, showed dynamic expression during fruit body formation [48,49,50]. During fruit body formation, expression divergence of wood-decay enzymes-related oxidation-reduction genes could be associated with their adaptation/colonization to the respective ecological niches by Pt and Pe, which may in turn further contribute to their morphological differences due to differences in nutritional source.

In conclusion, this study establishes that DNA methylomes of the two *Pleurotus* species, *P. tuoliensis* and *P. eryngii*, are highly conserved and largely static during development, while transcriptomes are dynamic. For a subset of genes, however, CG methylation may occur in promoter and/or body regions, and which primarily plays a repressive role on expression in both mushroom species.

## Supplementary Materials

The following are available online at https://www.mdpi.com/2073-4425/10/6/465/s1, Figure S1: Morphology of *Pleurotus tuoliensis* (Pt) and *P. eryngii* var. *eryngii* (Pe), Figure S2: Distribution of DMR in different categories of genomic features of Pt and Pe, Figure S3: Correlation of expression changes of ortholog sets between Pt and Pe at each phase, Figure S4: mCG and expression levels in transposable elements (TEs) regions of Pe, Figure S5: mCG difference and expression difference of differentially methylated TE (dmTE) between adjacent stages in Pt and Pe, Figure S6: mCG and expression levels in genic regions of Pe, Figure S7: mCG difference (in promoter region and gene body) and corresponding expression difference between adjacent stages in Pt and Pe, Figure S8: mCG divergence between promoter regions of orthologs and corresponding expression divergence in Pt and Pe, Figure S9: mCG divergence between gene bodies of orthologs and corresponding expression divergence in Pt and Pe, Table S1: Differentially methylated regions (DMRs) between adjacent developmental stages in both Pt and Pe, Table S2: Correlation of expression level between biological replicates and between species in each stage of both Pt and Pe, Table S3: GO enrichment of orthologs with divergence of expression changes in both phases, Table S4: Differentially methylated transposon elements (dmTEs) between adjacent developmental stages in both Pt and Pe, Supplementary dataset: TPM value of Pt and Pe in stages of mycelium (M), primordium (P) and fruit body (F).
