# Supplementary material for: Transcriptome Changes during Major Developmental Transitions Accompanied with Little Alteration of DNA Methylome in Two Pleurotus Species
Source: Genes (Basel). 2019 Jun 17;10(6):465. doi: 10.3390/genes10060465 (PMC6627472; doi:10.3390/genes10060465)
Supplement: Supplementary file 1 [file genes-10-00465-s001.zip › genes-497634-SI/Supplementary information.pdf]

Supplemental Information

Table S1. Differentially methylated regions (DMRs) between adjacent developmental stages in both *Pleurotus tuoliensis* (Pt) and *P. eryngii* var. *eryngii* (Pe)

	phase	hyper-methylated	hypo-methylated
Pt	phase1	84	96
	phase2	227	31
Pe	phase1	227	144
	phase2	775	653

Table S2. Correlation of expression level between biological replicates and between species in each stage of both *Pleurotus tuoliensis* (Pt) and *P. eryngii* var. *eryngii* (Pe)

	replicates among Pt	Replicates among Pe	Pt vs. Pe
mycelium	0.98	0.97	0.86
primordium	0.98	0.96	0.80
fruit body	0.98	0.96	0.76

Table S3. GO enrichment of orthologs with divergence of expression changes in both phases

	GO name	GO id	Gene Ratio	Bg Ratio	q-value
Phase1	oxidation-reduction process	GO:0055114	125/991	258/3020	0.000013
Phase2	oxidation-reduction process	GO:0055114	115/929	258/3020	0.00024

Table S4. Differentially methylated transposon elements (dmTEs) between adjacent developmental stages in both Pt and Pe

	phase	no change	hyper-methylated	hypo-methylated
Pt	phase1	2460	14	5
	phase2	2467	10	2
Pe	phase1	2835	22	14
	phase2	2710	70	91

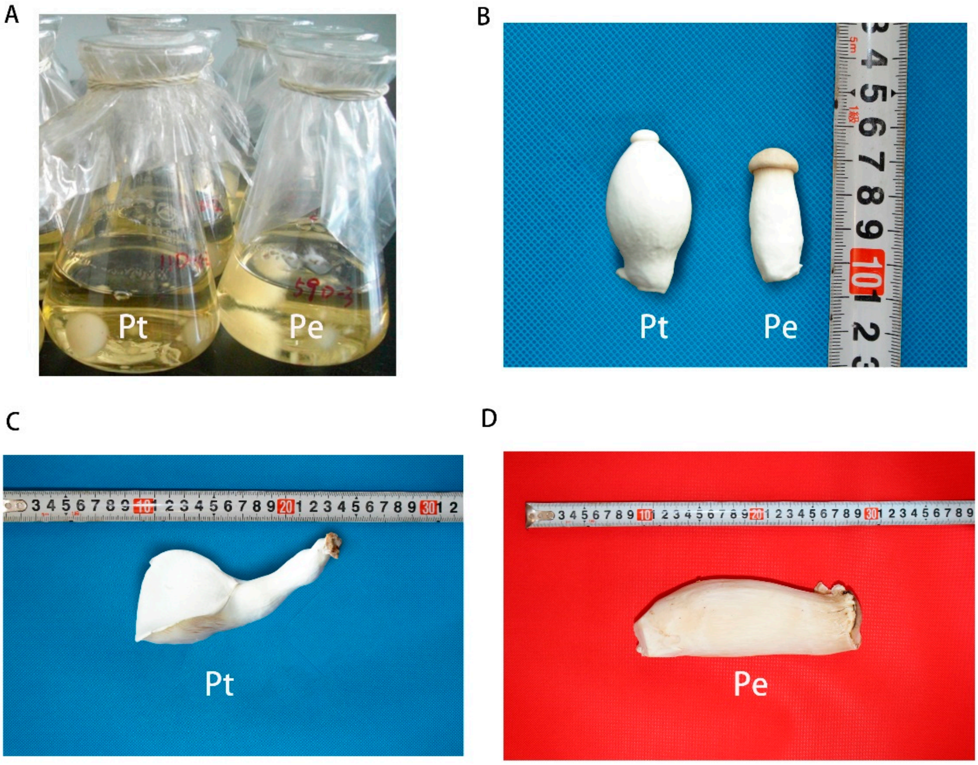

Figure S1 Morphology of *Pleurotus eryngii* sub. *tuoliensis* (Pt) and *P. eryngii* var. *eryngii* (Pe). (A) monokaryotic mycelium (B) primordium (C) fruit body of Pt and (D) fruit body of Pe.

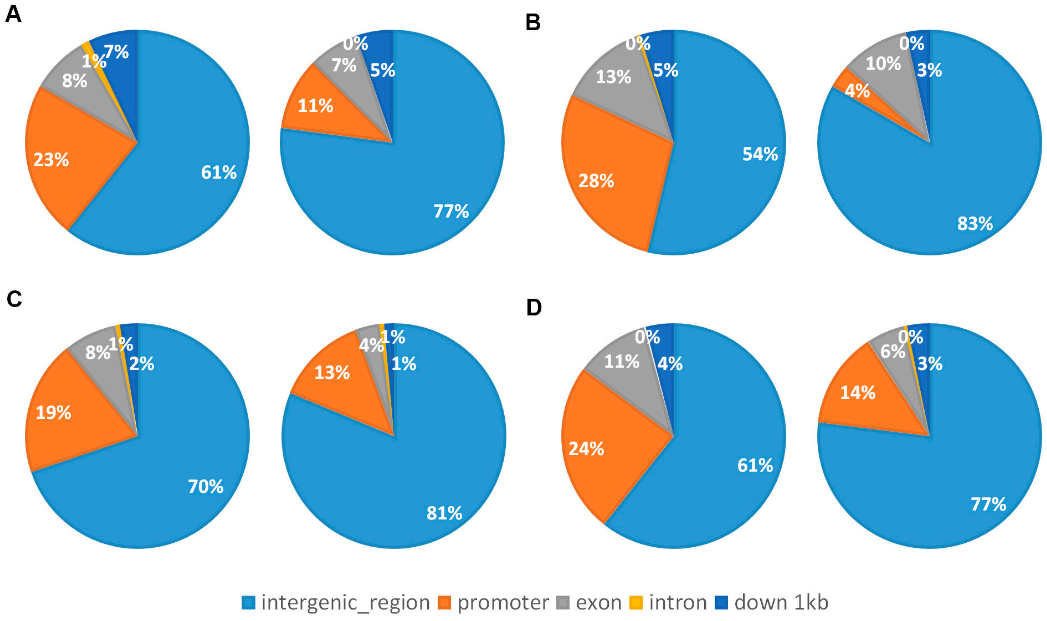

Figure S2. Distribution of DMR in different categories of genomic features of *Pleurotus tuoliensis* (Pt) and *P. eryngii* var. *eryngii* (Pe). (A, B) DMRs in phase1 (A) and phase2 (B) of Pt. (C, D) DMRs in phase1 (C) and phase2 (D) of Pe. For each subfigure, left and right panel represent hyper-methylated DMRs and hypo-methylated DMRs, respective. Number of DMRs in each phase are shown in Table S1.

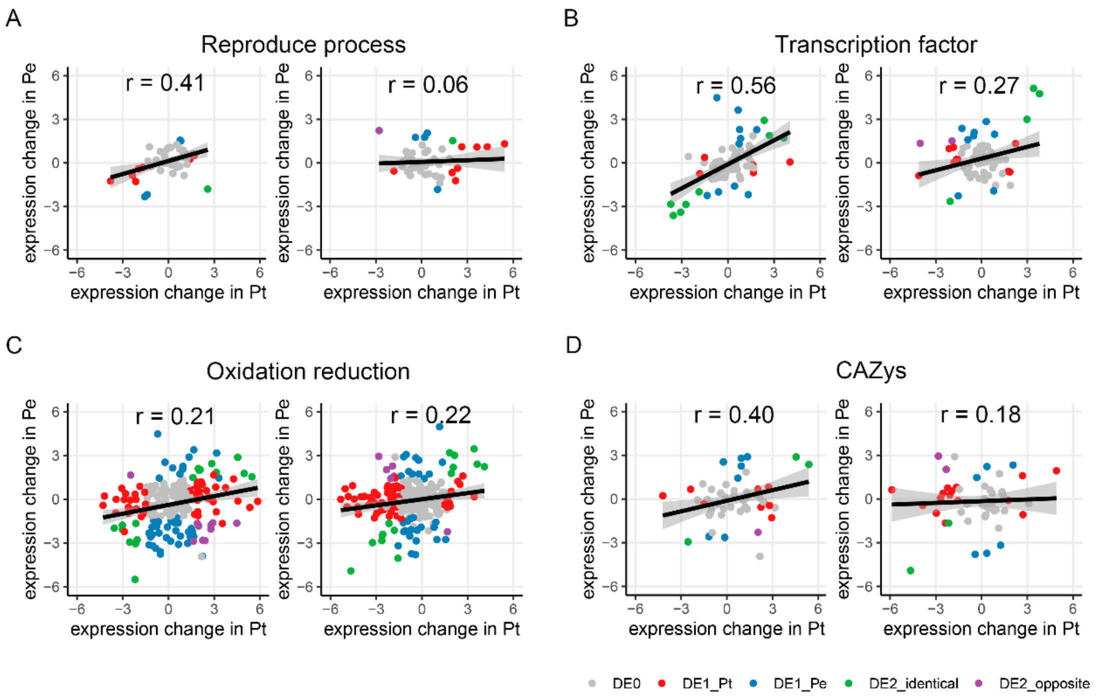

Figure S3 Correlation of expression changes of ortholog sets between *Pleurotus tuoliensis* (Pt) and *P. eryngii* var. *eryngii* (Pe) at each phase. (A) Reproduce process. (B) Transcription factor. (C) Oxidation reduction and (D) CAZys. The x-axis represents log2-transformed fold change between adjacent stages in Pt; the y-axis represents log2-transformed fold change between adjacent stages in Pe.

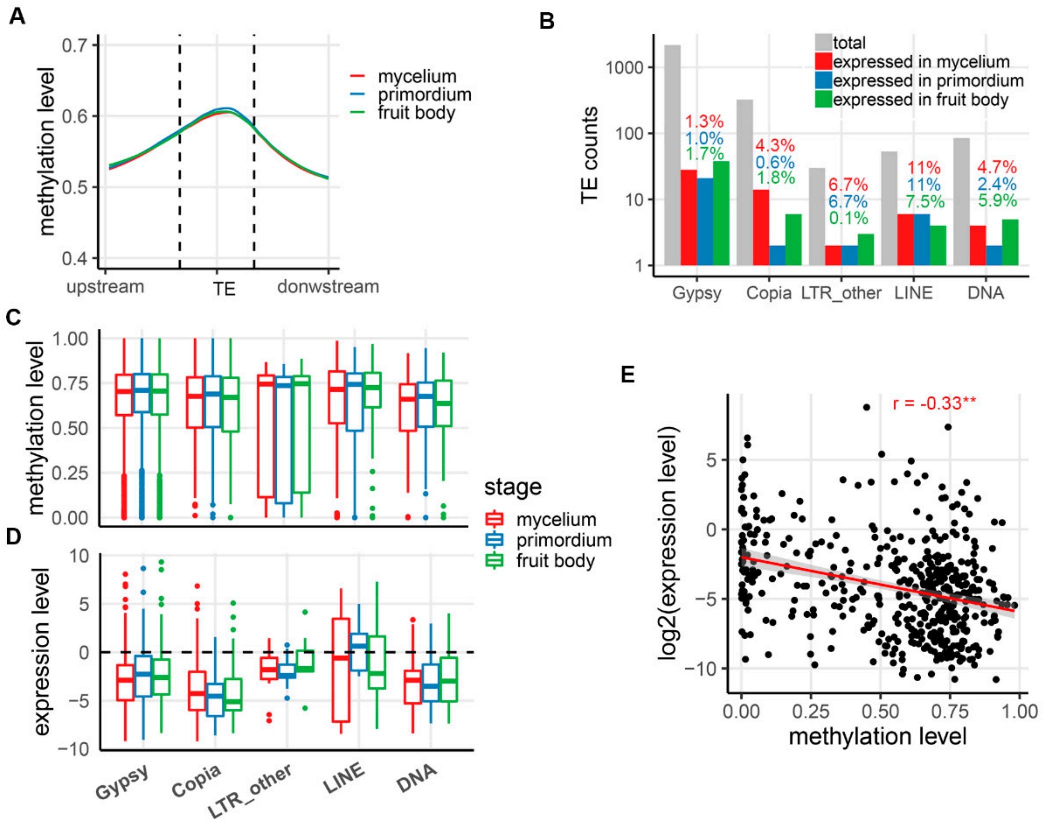

Figure S4. mCG and expression levels in transposable elements (TEs) regions of Pe. (A) Meta-plots of mycelium (MY, red), primordium (PR, blue) and fruit-body (FB, green) mCG levels in TEs. (B) Distribution and relative proportion of different classes of TEs. (C) Number of TEs in each class (grey bars) and number of expressed TEs (TPM > 1) in the MY, PR and FB stages. Percentages above the bars showing proportions of expressed TEs in each class. (D, E) mCG levels (D) and expression levels (E) for each class of TEs in the M, P and F stages. The dashed line in (E) indicates the static expression trend. The letters above the boxes denote results of Post Hoc Multiple Comparisons among the different developmental stages, wherein different letters denote statistical significance. Only TEs with a TPM value > 0 are shown in (E). (F) Negative correlation between mCGs and expression of TEs. Only TEs with a TPM value > 0 are tabulated.

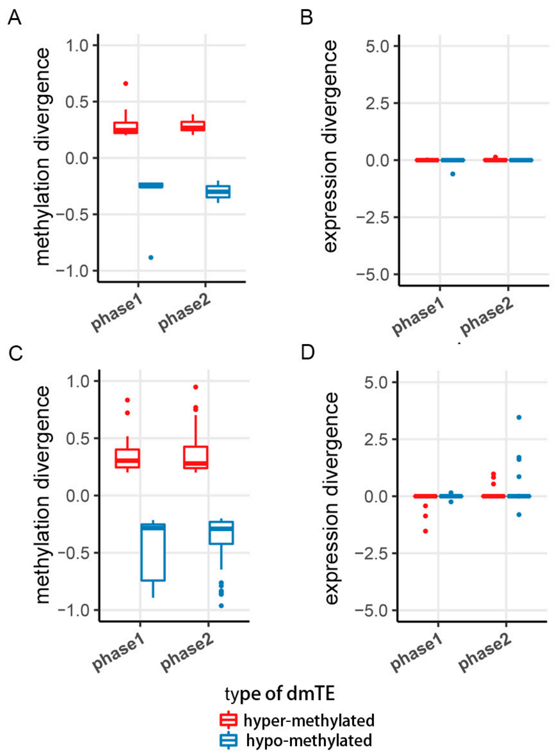

Figure S5. mCG difference and expression difference of differentially methylated TE (dmTE) between adjacent stages in Pt and Pe. (A, B) mCG (A) and expression (B) divergence in phas1 and phase2 of Pt. (C, D) mCG (C) and expression (D) divergence in phas1 and phase2 of Pe. mCG divergence is measured by difference value between adjacent stages, whereas expression divergence is measured by log-transformed fold change of expression level between adjacent stages. Number of dmTEs in each phase are shown in Table S4.

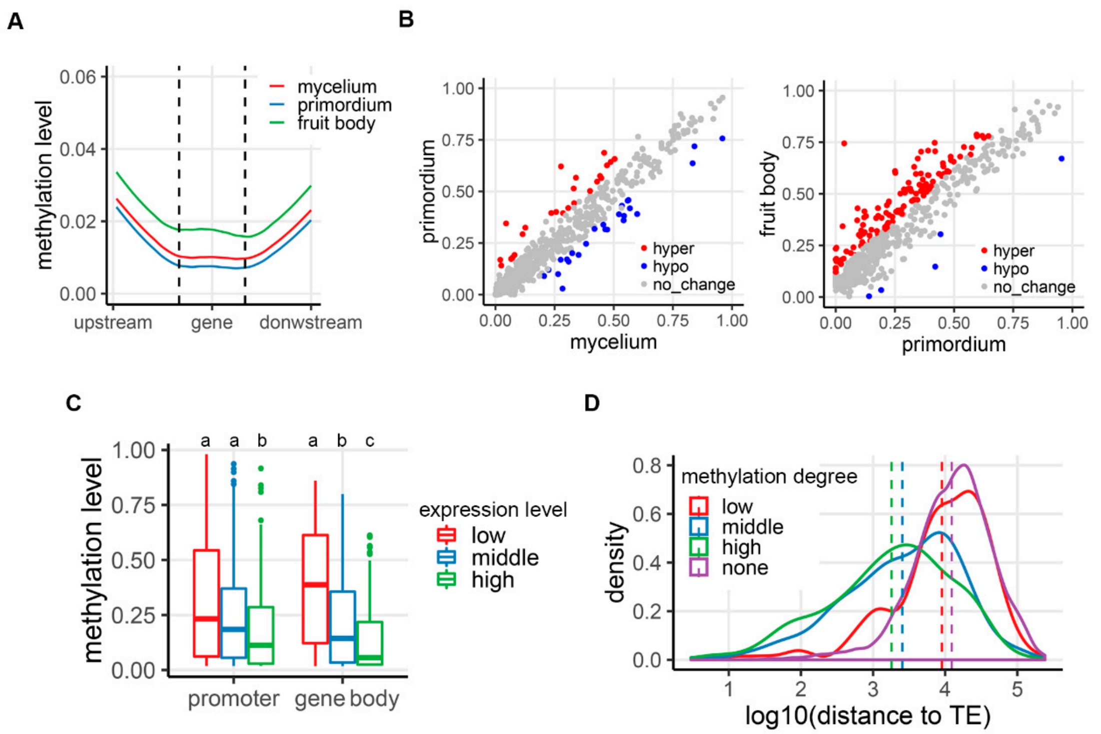

Figure S6. mCG and expression levels in genic regions of Pe. (A) Meta-plots of mycelium (M, red), primordium (P, blue) and fruit-body (F, green) methylation levels in genic regions. (B) Correlation of mCG levels in promoter regions during transition 1 (from mycelium to primordium, left panel) and transition 2 (from primordium to fruit-body, right panel). The red, blue and grey dots indicate hyper-methylated, hypo-methylated and unmethylated promoters, respectively. (C) Effects of mCG in promoters and gene bodies on gene expression (genes harboring methylated promoters or gene bodies were divided to low, medium and high expression classes). (D) Distribution of distances between different types of promoters (with respect to mCG) to their nearest TEs (none, low, medium, high mCG-containing promoters).

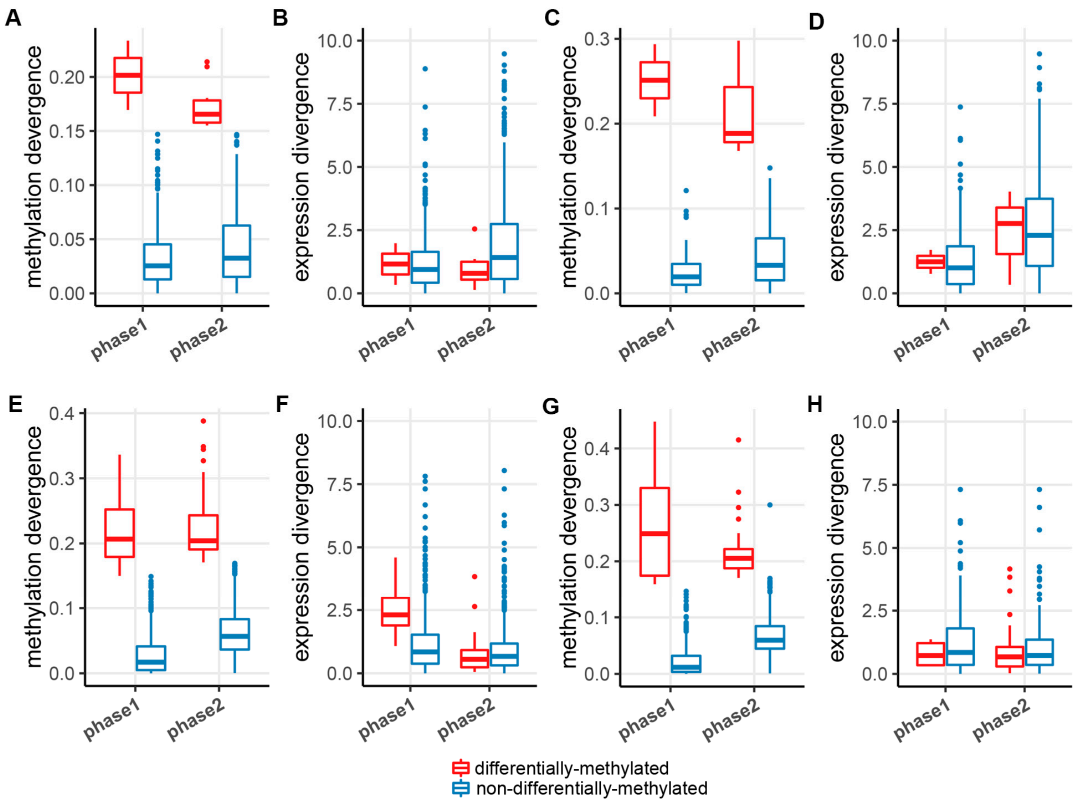

Figure S7. mCG difference (in promoter region and gene body) and corresponding expression difference between adjacent stages in Pt and Pe. (A, B) mCG difference of promoter region (A) and expression changes of corresponding genes (B) in Pt. (C, D) mCG difference of gene body (C) and corresponding expression changes (D) in Pt. (E, F) mCG difference of promoter region (E) and expression changes of corresponding genes (F) in Pe. (G, H) mCG difference of gene body (G) and corresponding expression changes (H) in Pe.

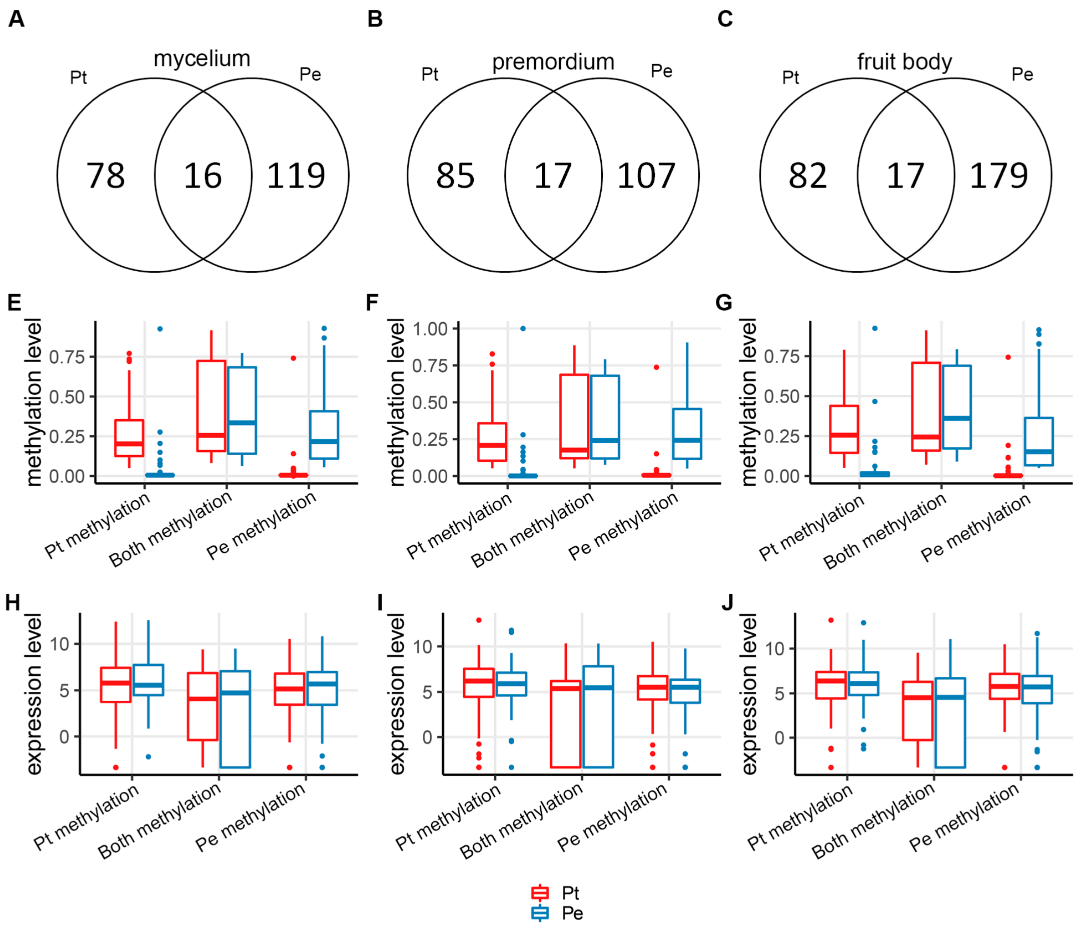

Figure S8. mCG divergence between promoter regions of orthologs and corresponding expression divergence in Pt and Pe. (A, B, C) Overlaps of methylated promoter (MP) between Pt and Pe among mycelium (A), primordium (B) and fruit body (C). (DEF) mCG divergence of Pt-specific MPs, Pe-specific MPs and overlapped MPs in mycelium (D), primordium (E) and fruit body (F). (H, I, G) expression divergence related to Pe-specific MPs, Pe-specific MPs and overlapped MPs in mycelium (H), primordium (I) and fruit body (J).

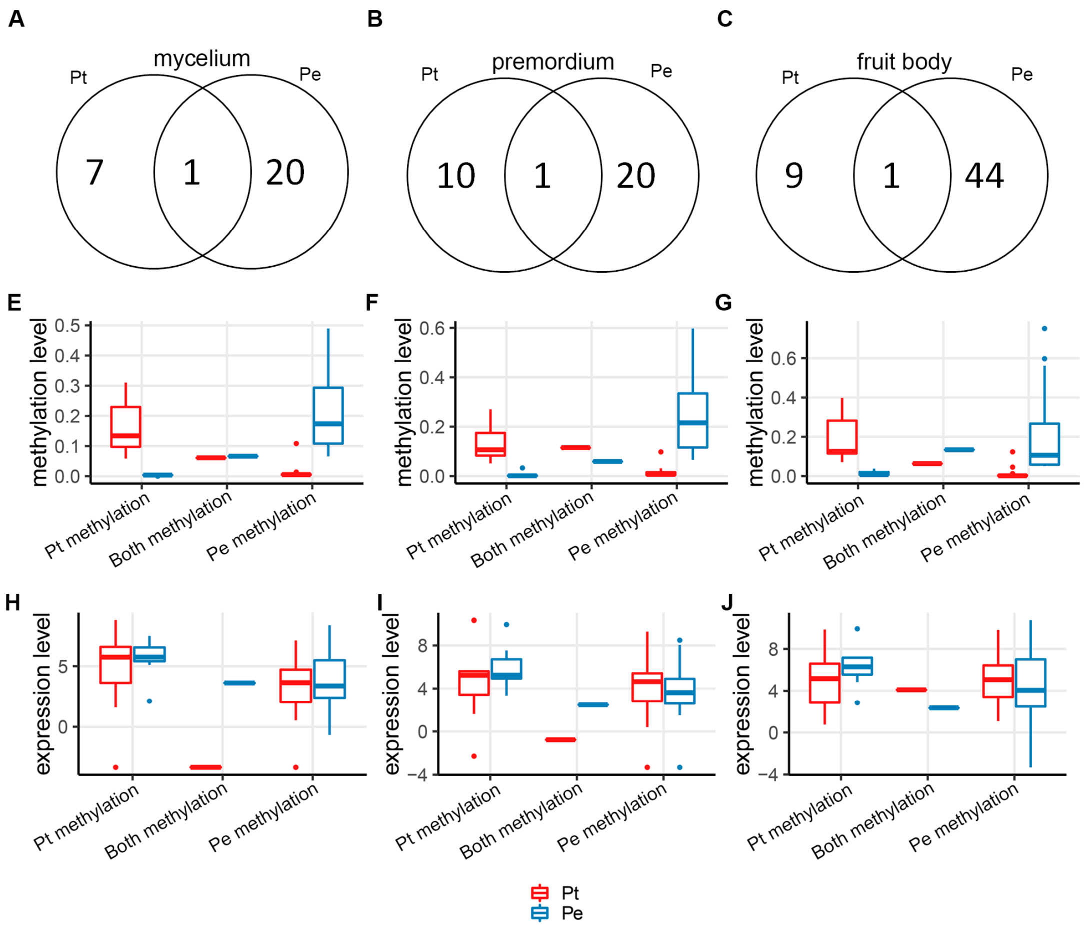

Figure S9. mCG divergence between gene bodies of orthologs and corresponding expression divergence in Pt and Pe. (A, B, C) Overlaps of methylated gene body (MGB) between Pt and Pe among mycelium (A), primordium (B) and fruit body (C). (DEF) mCG divergence of Pt-specific MGBs, Pe-specific MGBs and overlapped MGBs in mycelium (D), primordium (E) and fruit body (F). (H, I, G) expression divergence related to Pe-specific MGBs, Pe-specific MGBs and overlapped MGBs in mycelium (H), primordium (I) and fruit body (J).
